# Nasopharyngeal Tuberculosis: Epidemiology, Mechanism of Infection, Clinical Manifestations, and Management

**DOI:** 10.1155/2016/4817429

**Published:** 2016-02-29

**Authors:** Chonticha Srivanitchapoom, Pichit Sittitrai

**Affiliations:** ^1^Otolaryngology Unit, Phayao Hospital, Phayao 56000, Thailand; ^2^Department of Otolaryngology, Faculty of Medicine, Chiang Mai University, Chiang Mai 50200, Thailand

## Abstract

Nasopharyngeal tuberculosis (NPTB) is a noteworthy disease especially in its worldwide spread of the* Mycobacterium* infection. Although NPTB has been identified in less than one percent of TB cases, recent multiple case reports indicate an either increased awareness or incidence of this disease. The most helpful diagnostic tool is an uncomplicated nasopharyngeal biopsy. However, NPTB is usually ignored because it has varied clinical manifestations and similar presentations with other more common head and neck diseases. Furthermore, the most common presenting symptom is cervical lymphadenopathy mimicking nasopharyngeal carcinoma, a more common and serious disease. Treatment outcomes of NPTB are good in both HIV-positive or HIV-negative patients. In addition, pulmonary tuberculosis association was reported in wide range between 8.3% and 82% which should be considered in a treatment program. In conclusion, early diagnosis and management in NPTB can be achieved by (1) increased awareness of this disease, (2) improvement in knowledge regarding clinical manifestations, and (3) improvement of diagnostic techniques.

## 1. Introduction

Tuberculosis (TB) has remained one of the world's deadliest communicable diseases [1]. It can affect the lung (pulmonary TB) and other sites (extrapulmonary TB) as well. According to the WHO 2014, 6.1 million cases of TB were reported with 5.7 million newly diagnosed cases and 0.4 million previously diagnosed cases [1]. In new cases, 0.8 million patients had extrapulmonary TB [1]. Head and neck regions can be present in up to 10% of all TB cases with cervical lymph nodes most commonly involved [2]. In 1974, Rohwedder [3] reported 16 (1.8%) upper respiratory tract TB cases among 843 TB patients. Nasopharyngeal tuberculosis (NPTB) was identified less than 1% in the head and neck TB category [2, 3]. Previous literatures usually presented single case reports of NPTB; however, recently more large case series have been observed. This review summarized the epidemiology, mechanism of infection, clinical manifestations, diagnostic techniques, and management and also emphasized the association between NPTB and pulmonary TB.

## 2. Epidemiology

Prior to the introduction of chemotherapy, 1.4% of adenoids [4] and 6.5% of tonsils [5] removed from asymptomatic patients were infected by tubercle bacillus. Mahindra et al. [6] found adenoid TB in 18 out of 67 patients with TB cervical adenitis who underwent adenotonsillectomy. With the advent of effective antituberculous therapy and pasteurization of cow's milk, worldwide prevalence of this phenomenon appears to be reduced [7, 8]. Literature was searched from the PubMed database by utilizing the key word “nasopharyngeal tuberculosis.” Between August, 1947, and April, 2015, case reports of NPTB were noted in over 60 cases from both English and non-English articles. Large series studies were frequently reported from Asia (Table 1) [9–18]. There was a slight predominance of woman in all large series studies [10, 11, 13–15, 18]. NPTB commonly occurred in adults with varied average ages in each report. Four large series studies indicated a mean age between 30 and 40 years [10, 11, 13, 14] while two peaks of frequency were estimated, between 15 and 30 years and 50 and 60 years [19, 20]. Markham [21], however, reported three of the youngest patients who were 11, 13, and 15 months old from their adenotonsillectomy specimens.

According to the WHO global TB report, the most common coexisting condition for all TB patients was HIV infection which had an influence on treatment outcomes [1]. But in NPTB, only some of articles mentioned this relationship with good treatment outcomes in all articles whether HIV-positive or HIV-negative [11, 14, 22–24]. In addition, NPTB may mimic nasopharyngeal carcinoma (NPC) [17, 25] or coexist with other conditions in the head and neck such as laryngeal TB, oropharyngeal TB, and postradiation of NPC [22, 23, 25, 26].

## 3. Mechanism of Infection

In 1981, Innes [27] suggested six mechanisms whereby extrapulmonary TB might arise: (1) nonpulmonary primary infection, (2) lymphatic spread from a pulmonary primary complex, (3) haematogenous spread from a primary complex, (4) haematogenous spread from a postprimary lesion, (5) contiguous spread from organ to organ, and (6) epithelial implantation. However, for NPTB, both a primary nasopharyngeal infection which was defined as an isolated tuberculous infection of the nasopharynx in the absence of pulmonary or systemic diseases [11, 24] and a secondary spread via haematogenous or lymphatic system were described [11, 13, 14, 20, 28]. The rich lymphatic network of Waldeyer's ring explains lymphatic nasopharyngeal contamination [6, 11, 19, 20]. Swart et al. [29] indicated primary nasopharyngeal infection as droplet transfer due to this area being an impact area for inhaled air which was similar to other published articles [10, 13, 17]. While three large series studies supported theoretical secondary infection from the lymphatic system with a high percentage of pulmonary TB association [11, 13, 14], the association of pulmonary TB was observed in a wide range between 8.3% and 55.6% [10, 11, 13, 14, 16, 17]. Furthermore, Graft [28] reported that the highest prevalence of pulmonary TB associated with NPTB was 82% in his study. In summary, both mechanisms of infection were possible depending on the percentage of the association between NPTB and pulmonary TB in each study.

In addition, Chan et al. [25] described the mechanism of NPTB developed in postradiotherapy of the NPC. This may be partially due to local damage to the nasopharynx which achieved a high dose of radiotherapy (60 to 74 Gy), resulting in a breakdown of the mucosal barrier and a localized immunodeficiency or susceptibility.

## 4. Clinical Manifestations

NPTB can develop in a healthy patient without underlying disease and no history of tuberculosis contact nor an immune-compromised host [6, 24]. The clinical presentations can be systemic or nasopharyngeal symptoms. Common complaints are malaise, low grade fever, elevated temperature in the evening, repeated cold, night sweat, weight loss, and productive cough [6, 13, 14, 17, 19, 20]. The most common symptom is cervical lymph node enlargement which was observed more than 70% in many studies [10, 11, 13, 14, 17, 25]. The pattern of nodal enlargement in NPTB was different from primary cervical tuberculous lymphadenitis; the latter usually involved supraclavicular and posterior group also presented in unilaterally, multiple, or matted lymphadenopathy [30–33], while the former was affected along nasopharyngeal lymphatic drainage [10, 11, 14]. A retropharyngeal lymph node is a first echelon node and most commonly affected firstly and was identified from imaging [10]. However the upper jugular, middle jugular, and posterior group of cervical lymph nodes were more commonly found on physical examination, respectively [10, 11, 14]. Nodal features usually manifested as multiple and bilateral involvement (Table 2) [10, 11, 13, 14, 17].

The nasopharyngeal symptoms which could be presented alone or associated with nodal enlargement included (1) ear problems: aural fullness, hearing loss, otorrhea, otalgia, tinnitus, and middle ear effusion [34–36], (2) nose problems: running nose, postnasal drip, nasal obstruction, and epistaxis [25, 37, 38], (3) others: snoring, headache, and diplopia [10, 24, 39–41].

**Figure 1:**
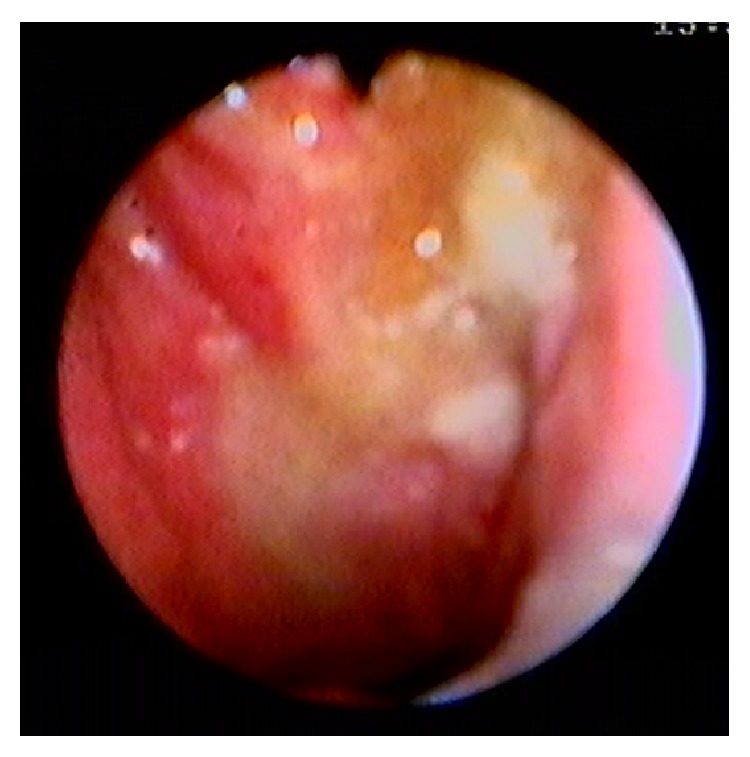
A 32-year-old woman, nasopharyngeal examination shows irregular redness mucosa and white patch covers the nasopharyngeal area.

On nasopharyngoscopic examination, different appearances on nasopharyngeal findings can be detected consisting of normal appearance, irregular mucosa, ulcerative lesion, mass, bulging or swelling, white patch cover the nasopharyngeal area (Figure 1), and lymphoid hyperplasia (Table 3) [11, 13, 14, 17].

## 5. NPTB Associated with HIV Infection

TB and HIV infection has been reported as coexisting and coepidemic condition. Thirteen percent of people who developed TB were HIV-positive. Treatment outcomes were worse for HIV-positive TB patients (74%) compared with HIV-negative patients (88%) and mortality was more than three times higher among HIV-positive TB patients [1]. Although the relationship between NPTB and HIV infection has not been clarified yet, few articles mentioned good treatment outcomes in both HIV-positive and HIV-negative NPTB patients [11, 14, 22–24]. However, HIV testing should be performed in every TB cases for reducing the burden of HIV-associated TB patients.

## 6. Diagnostic Techniques

### 6.1. Imaging

**Figure 2:**
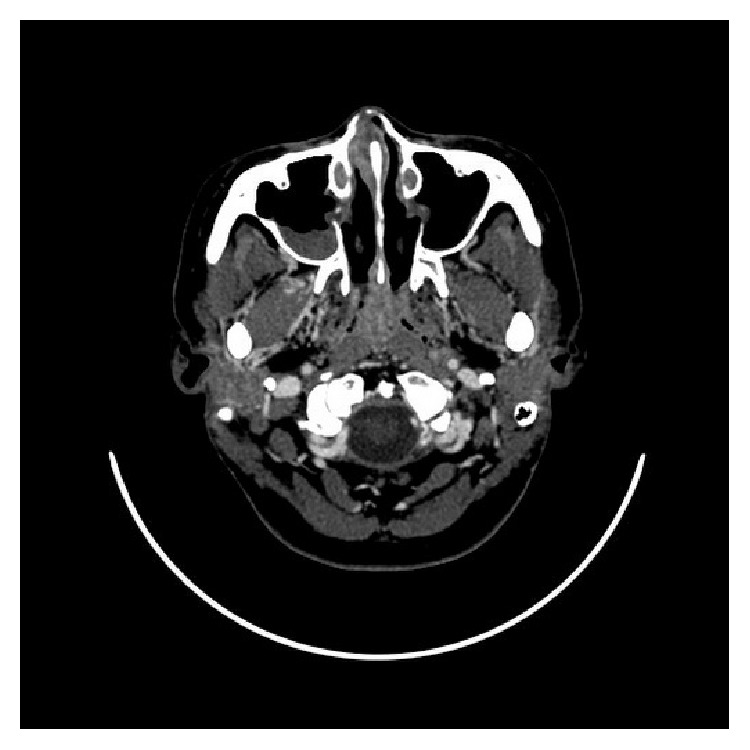
A 32-year-old woman, axial contrast-enhanced CT shows diffuse mucosal thickening without invasion into surrounding structures.

**Figure 3:**
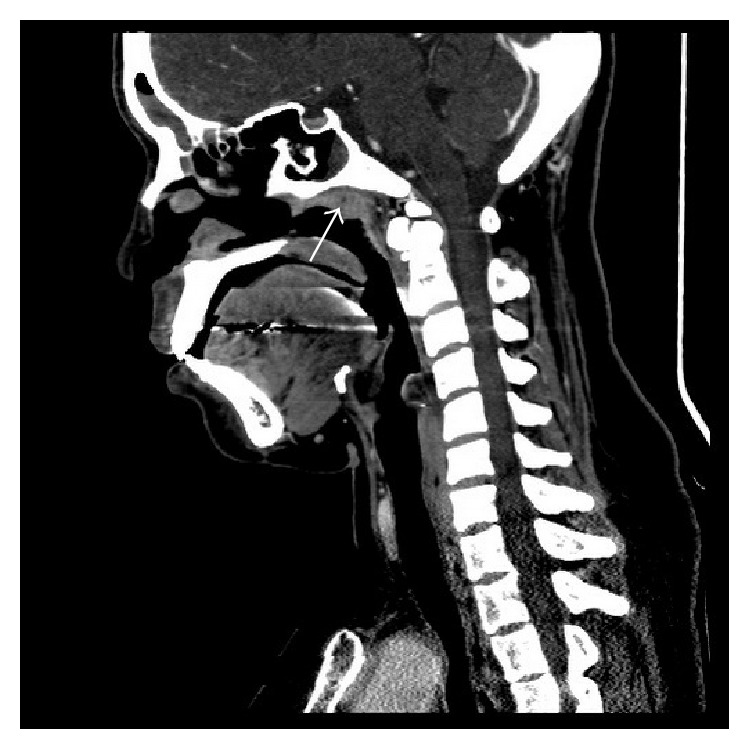
A 32-year-old woman, sagittal contrast-enhanced CT shows small central necrosis area in the nasopharyngeal lesion.

Computed tomography (CT) and magnetic resonance imaging (MRI) were reported as valuable tools in head and neck TB, demonstrating the sites, pattern, and extension of the disease [42, 43]. In addition, finer details of lesion detected from MRI imaging were helpful in distinguishing nasopharyngeal diseases [42]. Both nasopharynx and lymph nodes reveal their own suspected characteristics (Table 4) [10, 44]. Nasopharyngeal polypoid mass was the common finding on CT/MRI followed by diffuse mucosal thickening [10, 44]. The lesion was usually confined in the nasopharyngeal area without invasion into the surrounding structures such as skull base, prevertebral muscle, nasal cavity, and oropharyngeal area (Figure 2). Cai et al. [10] suggested that a small necrosis in a nasopharyngeal lesion which was caseous necrosis on pathology might be a valuable clue to diagnose NPTB (Figure 3), although the area of necrosis might have a similar appearance on NPC but was seen more often in a large tumor or after treatment [10]. King et al. [44] mentioned that TB which involved lymphoid tissue sometimes causes destruction of the normal lymphoid architecture. Thus a striped feature which is a recognized feature of benign enlargement of the nasopharyngeal adenoid can cause destruction. These points might be helpful in distinguishing between NPTB and adenoids [10, 44]. Cervical lymphadenopathy also detected from imaging had their own different features according to the stage of nodal disease (Table 4) [10, 44].

Recently, 18F-FDG PET/CT has become an established imaging tool in oncology and is now being applied to the field of infection and inflammatory diseases [42, 45, 46]. The advantages for NPTB include (1) clearly indicated locations of TB lesions [42]; (2) identification of extrapulmonary TB [42, 45]; (3) a metabolic abnormality which guided biopsy sites [42]; (4) assessment of treatment response [45, 46]. In addition, 18F-FDG PET had its own ability to distinguish active from inactive disease in pulmonary TB by dual time point imaging [45]. However, standardized uptake value measurements are high in both TB and malignant lesions, with significant overlap that limits their usefulness [42, 45].

### 6.2. Tissue Diagnosis

The gold standard for diagnostic TB was a positive* Mycobacterium *spp. bacterial culture either from tissue or from sputum that also achieves drug sensitivity [2, 32, 33, 44]. But this test required a waiting period from four to six weeks [20, 32, 33]. Histopathology was suggested to be a helpful diagnostic tool. NPTB gross lesions can mimic other nasopharyngeal treatable diseases including NPC, lymphoma, minor salivary gland carcinoma, Wegener's granulomatosis, angiofibroma, fungal infection, sarcoidosis, periarteritis nodosa, leprosy, syphilis, and Castleman's disease [22, 25, 26, 34, 38, 44, 47]. A typical pathological report for diagnosing NPTB is caseating granulomatous inflammation with multinucleated giant cells of Langhans' type and foreign body giant cells, with or without necrosis [25, 32, 33], although NPC itself can develop a granulomatous reaction, similar to TB, in peritumoral tissue [25, 44]. Furthermore chronic granulomatous inflammation with positive Ziehl-Neelsen staining for acid-fast bacilli or bacterial culture can also be demonstrated [11, 14, 33, 44]. In addition, the case which is strongly suspicious for TB but negative for bacterial culture, bacterial stain, and polymerase chain reaction (PCR) analysis for* M. tuberculosis* DNA is helpful [2, 22, 24, 25, 32]. Many authors suggested that Ziehl-Neelsen staining for acid-fast bacilli in a histologic section is reliable and sensitive and is faster and less costly than bacterial culture and PCR analysis. The latter method should be reserved for the stain negative cases to avoid missing a treatable, potentially lethal infection [13, 20, 22, 24, 25].

## 7. Management

The minimal duration of extrapulmonary TB treatment is six months [11, 14, 19, 20]. Treatment regimen is either a triple combination including isoniazid (INH), rifampicin (RFP), and ethambutol (EB) for 9–18 months or a quadritherapy which add pyrazinamide (PZA) for nine months [19, 20]. In addition, Waldron et al. [17] preferred a standard regimen of INH, RFP, and PZA for a minimum period of six months and added streptomycin during the first two to three months. Some authors preferred two months of INH, RFP, EB, and PZA followed by INH and RFP for four to seven months [11, 14, 22, 40]. With adequate medical treatment, nasopharyngeal tuberculosis carries a good prognosis and no cases of resistance to antituberculous drugs or therapeutic failure had been noticed [9, 11, 14, 17–20, 22, 40].

## 8. Conclusion

Although NPTB is uncommon in previous literature, recently an increased number of large case series have been reported. The suspected reasons include (1) awareness of the disease; (2) improvement of knowledge regarding clinical manifestation; (3) improvement of diagnostic techniques; and (4) increased incidence of the disease. According to the variability of clinical manifestations, awareness of this disease and precise evaluation of the patient were main considerations to avoid misdiagnosis. Especially differentiating between NPC and NPTB, the former has similar clinical presentations which include cervical lymph node enlargement and lesions in nasopharynx. Tissue diagnosis should be a consideration in all cases. Typical histopathology with caseating granulomatous inflammation usually must be identified. Also diagnosis must be confirmed by demonstrating acid-fast bacilli staining. However in highly suspicious case, bacterial culture and PCR analysis should be performed.

## Figures and Tables

**Table 1:** Summary of large series NPTB cases.

Authors	Country	Year	Sex M : W	Age (mean) (years)	Associated with pulmonary TB (*n* PTB/*n* total)	Number of cases
Jian et al. [9]^*∗*^	China	2012	NA	NA	NA	50
Cai et al. [10]	China	2013	15 : 21	17–68 (30.5)	31% (11/36)	36
Srirompotong et al. [11]	Thailand	2004	9 : 14	20–71 (38)	44.4% (8/18)	23
Wang and Zhu [12]^*∗*^	China	2013	NA	NA	NA	19
Tse et al. [13]	Hong Kong	2003	4 : 13	20–74 (39)	55.6% (5/9)	17
Chongkolwatana et al. [14]	Thailand	1998	7 : 8	7–65 (31.7)	36.4% (4/11)	15
Eng et al. [15]^*∗*^	Taiwan	1996	2 : 12	17–61 (NA)	NA	14
Su et al. [16]^*∗*^	China	2002	NA	NA	8.3% (1/12)	12
Waldron et al. [17]	Hong Kong	1992	5 : 5	25–76 (40.5)	10% (1/10)	10
Oudidi et al. [18]^*∗*^	Morocco	2007	2 : 4	NA (41)	NA	6

[reference number]^*∗*^ = article in non-English language and only abstract available.

NA = not available.

**Table 2:** Clinical characters of cervical lymphadenopathy in NPTB.

Authors	% of lymphadenopathy (*n* LN enlargement : *n* total)	Bilateral (%) : unilateral (%)	Multiple (%) : solitary (%)
Cai et al. [10]	80.6% (29 : 36)	65.5 : 34.5	79.3 : 20.7
Srirompotong et al. [11]	91.3% (21 : 23)	71.4 : 28.6	90.5 : 9.5
Tse et al. [13]	58.8% (10 : 17)	20 : 80	NA
Chongkolwatana et al. [14]	93.3% (14 : 15)	42.9 : 57.1	64.3 : 35.7
Waldron et al. [17]	70% (7 : 10)	57.1 : 42.9	71.4 : 28.6

**Table 3:** Nasopharyngeal findings from large 4 series.

Nasopharyngeal findings	Srirompotong et al. [11] (*n* = 23)	Tse et al. [13] (*n* = 17)	Chongkolwatana et al. [14] (*n* = 15)	Waldron et al. [17] (*n* = 10)
Normal	7 (30%)	1 (6%)	2 (13.3%)	2 (20%)
Irregular mucosa	5 (22%)	6 (35%)	7 (46.6%)	NA
Ulcerative	2 (9%)	1 (6%)	1 (6.7%)	NA
Mass	9 (39%)	6 (35%)	3 (20%)	4 (40%)
Bulging or swelling	NA	1 (6%)	1 (6.7%)	NA
White patch on mucosa	NA	1 (6%)	NA	NA
Lymphoid enlargement	NA	NA	1 (6.7%)	4 (40%)
No data	NA	1 (6%)	NA	NA

NA = not available.

**Table 4:** Summary of CT/MRI findings [10, 44].

Site	CT/MRI findings
Nasopharynx	Polypoid mass
	Diffuse thickening of the mucosal wall of nasopharynx
	Less extension outside the nasopharynx
	Less invasion into surrounding structures such as prevertebral muscle, nasal cavity, and oropharynx
	Lesion necrosis especially within small nasopharyngeal lesion
	Destruction of striped feature

Lymph node	Homogeneous contrast enhancement (early phase)
	Peripheral rim enhancement with central necrosis (progression phase)
	Fibrosis and calcification may have homogeneous appearance without enhancement (late phase)
	Shortest axial diameter ≥ 5 mm for retropharyngeal LN and ≥10 mm for LN of neck
